# Voluntary Wheel Running as Refinement Tool for Postoperative Severity Assessment and Humane Endpoint Detection in Rats with Brain Tumors

**DOI:** 10.3390/brainsci16060635

**Published:** 2026-06-13

**Authors:** Alina L. Ottlewski, Christine Häger, Elvis J. Hermann, Franck Fogaing Kamgaing, Mesbah Alam, Jannik D. Schwabe, Hauke Thiesler, Herbert Hildebrandt, Aylina Glasenapp, Marion Bankstahl, Steven R. Talbot, Joachim K. Krauss, Kerstin Schwabe

**Affiliations:** 1 Department of Neurosurgery, Hannover Medical School, Carl-Neuberg-Straße 1, 30625 Hanover, Germany; 2 Institute for Laboratory Animal Science, Hannover Medical School, Carl-Neuberg-Straße 1, 30625 Hanover, Germany; 3 Institute of Clinical Biochemistry, Hannover Medical School, Carl-Neuberg-Straße 1, 30625 Hanover, Germany; 4 Center for Systems Neuroscience Hannover (ZSN), Bünteweg 2, 30559 Hanover, Germany; 5 Department of Biological Sciences and Pathobiology, Pharmacology and Toxicology, University of Veterinary Medicine Vienna, 1210 Vienna, Austria

**Keywords:** voluntary wheel running, burden assessment, humane endpoint, intracranial tumor model, rat

## Abstract

Background: In rodent models of intracranial tumor development, evaluating the actual burden experienced by animals beyond procedural severity is essential for ethical and legal compliance. This study examined whether voluntary wheel running (VWR) could serve as a sensitive indicator of post-surgical burden following subcutaneous transmitter implantation, tumor cell injection, and tumor resection. It also assessed whether VWR supports the detection of humane endpoints. VWR outcomes were compared with body weight, clinical scores, heart rate, and activity levels recorded via telemetry. Methods: Fourteen male BDIX rats were housed individually in cages equipped with a running wheel. Under general anesthesia, telemetric devices to monitor heart rate and activity were subcutaneously implanted. After recovery, glioblastoma BT4Ca cells were stereotaxically injected into the right frontal cortex. Eight days later, the resulting tumors were microsurgically resected. Body weight, VWR, heart rate, and general activity were continuously monitored until the animals reached humane endpoint criteria, indicated by sudden weight loss and clinical deterioration. Results: On average, body weight and VWR declined significantly after all surgical procedures, with tumor resection causing the most pronounced effect. As animals approached the endpoint, a marked drop in these parameters was observed, along with an increased clinical score (*p* < 0.05). Activity measures supported these findings, though less consistently than weight and VWR. Conclusions: Monitoring body weight and VWR enables an effective assessment of the actual postoperative burden experienced by rats undergoing surgeries of different procedural complexity. Moreover, VWR is a valuable supplementary tool for identifying humane endpoints alongside body weight and clinical scoring.

## 1. Introduction

Severity assessment in laboratory animals is essential to improve welfare and minimize suffering. The EU Directive 2010/63/EU therefore mandates severity assessment of experimental procedures for project approval. Yet, very few objectively applicable and standardized parameters exist, particularly for the fine-grading of severity in scientific procedures. Moreover, routinely applicable, non- or minimally invasive approaches for objective and routine classification of postsurgical burden in neuroscience are limited.

Clinical scoring and body weight (BW) have long served as primary indicators of animal well-being in experimental settings. More recently, increasing importance has been placed on species-specific behaviors, such as burrowing or activity measures [1,2,3]. However, these methods require experienced observers or direct interaction, which can be problematic as rats tend to mask signs of pain or conceal a deteriorating general condition to avoid attracting predators [4]. Observer-independent methods, such as telemetry, are becoming popular because they enable contactless and unbiased monitoring of heart rate and activity in the home cage [5,6,7,8,9,10].

Voluntary wheel running (VWR) is a method used to assess activity and has been employed in the investigation of the effects of exercise on obesity, cardiovascular disease, and type 2 diabetes [11,12,13]. Also, long-term VWR is rewarding and has been shown to induce plasticity in the mesolimbic pathway [14]. It has also been used to determine pain-related mobility impairment [15,16]. In mice, VWR has been used to evaluate recovery following partial hepatectomy and surgical implantation of a telemetric device [17,18]. Clustering VWR and BW data has also enabled grading of severity in models of acute colitis and restraint stress models [19]. In rats, VWR has been used in the context of epilepsia [20] and frontal cortical contusions [21]. It has also been used to investigate well-being in models of depression, migraine and peripheral inflammatory pain [22].

In neuroscience research, complex intracranial procedures are employed, each of which is potentially associated with different levels of severity. This study used a rat model of intracranial glioma to evaluate the value of VWR in assessing the postoperative burden of complex neurosurgical procedures, i.e., stereotaxic tumor cell injection through a small burr hole, and tumor resection within the frontal brain tissue via a large craniotomy. This model has been used in our working group for years to investigate the local treatment of glioblastomas. It also requires the definition of humane endpoints that request a balance between sufficient pathology for disease progression and the obligation to minimize animal suffering. Additionally, subcutaneous implantation of a telemetric device allows monitoring of postoperative burden through heart rate and general activity, providing a physiological reference alongside VWR. Finally, we applied the composite score for relative severity assessment (RELSA) after different surgeries and at the endpoint [23] and entered the daily weight measures into the endpointR app [24]. In brief, this algorithm utilizes the daily collected BW to determine the animal’s weight fluctuations. In case a rat’s BW falls outside this individually calculated range, the app sends a warning signal, considering that the particular animal may be at risk of reaching the humane endpoint.

## 2. Materials and Methods

### 2.1. Animals and Husbandry

Experimentally naïve male BDIX rats (275–420 g BW) were obtained from the Central Animal Facility of Hannover Medical School. The planned sample size was determined a priori using G*Power (version 3.1) (effect size f = 0.4, α = 0.05, power 1 − β = 0.80), yielding a required group size of *n* = 11. Because several animals at different time points in the experiment were excluded from statistical analysis due to transmitter and/or running wheel failure, three additional rats were approved, resulting in a final cohort of *n* = 14. Only males were chosen because of the requirements in our animal facility room. Rats were single-housed in type IV Macrolon^®^ cages (Tecniplast, Varese, Italy) inside a Scantainer^®^ (Scanbur, Karlslunde, Denmark) in a room with controlled environmental conditions (22 ± 2 °C, relative humidity of 55 ± 5%, 14/10 h light/dark cycle with a dark phase from 8:00 p.m.). Bedding (Espentiereinstreu AB P3, AsBe-wood GmbH, Gransee, Germany) was changed weekly. Enrichment included paper towels, two wooden sticks, and a cardboard house. A standard pellet diet (Altromin, Lage, Germany) and tap water were served ad libitum. Health monitoring, adapted to [25], followed a sentinel system. Ethics approval: This study was conducted in accordance with German law for animal protection and the European directive 2010/63/EU, including approval by the Lower Saxony State Office for Consumer Protection and Food Safety (LAVES, license AZ 18/2837), also following the ARRIVE guidelines.

### 2.2. Study Design

To acclimatize the rats to the running wheel, they were kept in cages with a running wheel integrated into the lid for seven days, with free access to the wheel. Then, they underwent three surgical interventions. First, a telemetric device (transmitter) was subcutaneously implanted for contactless monitoring of heart rate and activity. After a 3-week recovery, BT4Ca glioblastoma cells were stereotaxically injected into the right frontal cortex (tumor cell injection).

**Figure 1:**
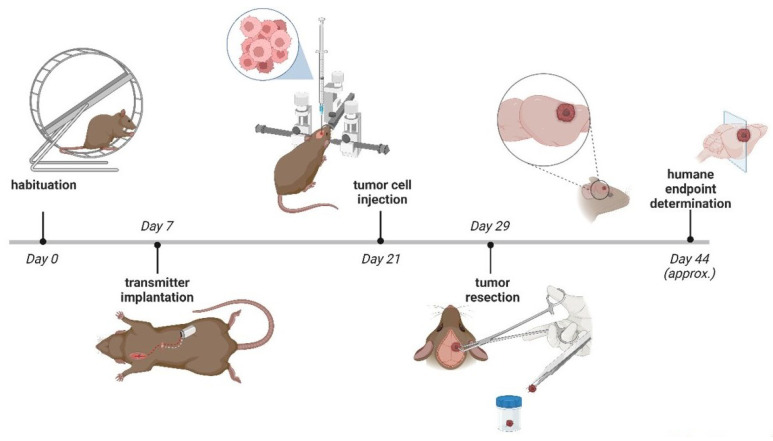
A schematic timeline outlining the key stages of the experimental study. It includes transmitter implantation, tumor cell injection, tumor resection, and humane endpoint determination. Prepared with BioRender.

On the 8th day after tumor cell injection, the developed tumors were microsurgically resected (tumor resection). Rats were sacrificed at humane endpoint determination, and the brains were histologically processed to verify tumor recurrence (Figure 1). The clinical score and BW were assessed daily throughout the study, alongside VWR, heart rate, and activity as indicators of well-being. Each rat served as its own control; baseline data obtained before surgery were compared with postoperative data. Blinding was not feasible because all animals underwent the same surgical procedures in a fixed sequential order, and the resulting visible scars indicated the respective surgical stage.

### 2.3. Surgical Interventions

#### 2.3.1. Transmitter Implantation

The transmitter was subcutaneously implanted under multimodal anesthesia. For perioperative systemic analgesia, carprofen (Rimadyl^®^ 50 mg/mL, Zoetis, Berlin, Germany) was 10 × diluted in 0.9% sterile sodium chloride (B. Braun, Melsungen, Germany) and subcutaneously injected with 5 mg/kg 30 min before surgery. Thereafter, all rats were intraperitoneally anesthetized with chloral hydrate (360 mg/kg in 10 mL/kg injection volume [3.6%]; Sigma-Aldrich, Darmstadt, Germany), which was combined with local anesthesia (a mixture of lidocaine [Xylocain 0.5%, AstraZeneca, Södertälje, Sweden] and bupivacaine [Carbostesin^®^ 0.25%, AstraZeneca, Södertälje, Sweden]) subcutaneously injected along the intended incision line (about 0.2 mL). The depth of anesthesia was controlled every few minutes with the foot pinch reflex, and chloral hydrate was re-administered at 1/3 of the initial dose when signs of anesthetic wear-off appeared. Eyes were protected with dexpanthenol-containing ointment (Bepanthen Augen-und Nasensalbe, 10 g, BAYER Vital GmbH, Leverkusen, Germany). Thereafter, the transmitter was implanted via a 2 cm skin incision into a subcutaneously prepared pocket on the animal’s left lateral side caudal to the costal arch (19 × 13 × 5 mm; ETA F10; PhysioTel Telemetry System, DSI, St. Paul, MN, USA). The electrocardiogram electrodes were subcutaneously tunneled to the right pectoralis major (negative electrode) and the xiphoid process (positive electrode) and secured with non-absorbable suture material. The incisions were closed with sutures and suture clips (see [9] for details).

Postoperatively, carprofen was applied via drinking water at 10 mg/kg/24 h for three days, considering the pre-surgical individual fluid intake. Blood was taken from the tail vein during the postoperative period to determine carprofen plasma concentrations [26].

#### 2.3.2. Intracranial Surgeries

Tumor cell injection: rats were anesthetized as described for transmitter implantation and placed in a stereotaxic frame. Five minutes after subcutaneous lidocaine injection along the incision line, a midline skin incision exposed Bregma as the reference point for target coordinates: +2.6 mm anterior, +2.5 mm lateral [27]. A small burr hole was drilled, and $10^{4}$ BT4Ca cells in 3 μL PBS were injected at 1 μL/min in a depth of 2.8 mm with a 10 μL Hamilton syringe (26 G needle, SGE Analytical Science, Ringwood, Australia; see [24] for details).

Tumor resection: eight days after tumor cell injection, a solid tumor is visible at the cortical surface and can be resected [24]. Rats were anesthetized and placed in a stereotaxic frame, and lidocaine was injected along the incision line. The midline skin incision was reopened, and a ∼2 mm craniotomy was drilled around the original burr hole to expose and microsurgically remove the tumor.

After tumor cell injection and tumor resection, the skin was closed with suture clips. Perioperative analgesia was provided during transmitter implantation.

### 2.4. Measures for Severity Assessment

Clinical scoring and BW: the general health score was assessed daily by an experienced scientist and veterinarian between 8:00 and 10:00 a.m. until humane endpoint detection according to the clinical score depicted in Table A1. After surgery, a score of 3 was temporarily accepted, as our experience shows that in this case, rats usually recover within 24 h. However, after recovery from tumor resection, sudden weight loss and mild ataxia (score 3) met the humane endpoint criteria upon suspected tumor recurrence, as previous studies have shown that in our glioblastoma model, this combination reliably predicts rapid deterioration and death, most likely within the following hours [24]. Weight was also entered into the EndpointR app daily [24]. In brief, this algorithm utilizes the daily collected BW to determine the animal’s weight fluctuations. If a rat’s BW falls outside this individually calculated range, the app sends a warning signal, as the particular animal may be at risk of reaching the humane endpoint. Thereupon, researchers and caretakers conducting the experiment check the animals at shorter intervals and decide to euthanize them whenever the clinical score deteriorates. Transmitter (1–2 g) and tumor weight (<100 μg) were not accounted for in the weight measures. Voluntary Wheel Running: each type IV Macrolon^®^ cage was provided with a running wheel (diameter 31.5 cm, 10 cm wide) integrated into the lid for free access. Wheel rotations per minute were continuously recorded using external light sensors and stored on a micro-SD card via an Arduino. Transmitter: heart rate and activity were measured continuously via the Ponemah 6.41 (PhysioTel Telemetry System DSI, St. Paul, MN, USA).

### 2.5. Statistical Analysis

All graphs were created using GraphPad Prism 10 (GraphPad Software, San Diego, CA, USA, 2023) and displayed as mean ± SEM, including individual values. Statistical analyses were two-sided, with significance set at *p* < 0.05 (SigmaPlot 15, Systat Software, San Jose, CA, USA). Normal distribution was assessed using a Shapiro–Wilk test. Thereafter, repeated measures (RM) ANOVA was used to assess changes in BW, VWR, heart rate, and activity after each surgery, respectively, before the humane endpoint. A one-way ANOVA was used for comparison between interventions, followed by adequate post hoc testing in case of significance. In addition, we applied the RELSA [23] score to compare the relative welfare of different surgeries and the humane endpoint. This algorithm-based comprehensive composite score detects relative welfare impairments from multi-dimensional input parameters of physiology and behavior. It achieves relative comparability by expressing the multidimensional change in the differences between the input variables and a reference set with a fixed severity quality. In this study, the reference set was obtained through the implantation of the transmitter and the injection of tumor cells. Additionally, the time-independent maximum RELSA score (RELSA_*max*_) in each animal was used to compare different surgical approaches and endpoints quantitatively. Finally, we used the RELSA to present the state of severity over time (RELSA_*flow*_) or the most pronounced severity displayed by the animals throughout the procedure (RELSA_*max*_). To compare the severity of surgical approaches and endpoints with RELSA, we included BW and VWR as input variables to illustrate the severity course over time (RELSA_*flow*_) and to compare the peak severity experienced by each animal (RELSA_*max*_; [23]). Only animals for which complete weight and running wheel data were available for the various interventions were used for this analysis.

### 2.6. Histology

Upon humane endpoint detection, rats were euthanized (720 mg/kg of intraperitoneal chloral hydrate [7.2%]). After respiratory arrest, rats were transcardially perfused with 4% paraformaldehyde, and their brains were sectioned and Nissl-stained (Thionine) to verify tumor recurrence.

## 3. Results

### 3.1. Postoperative Phase

**Figure A1:**
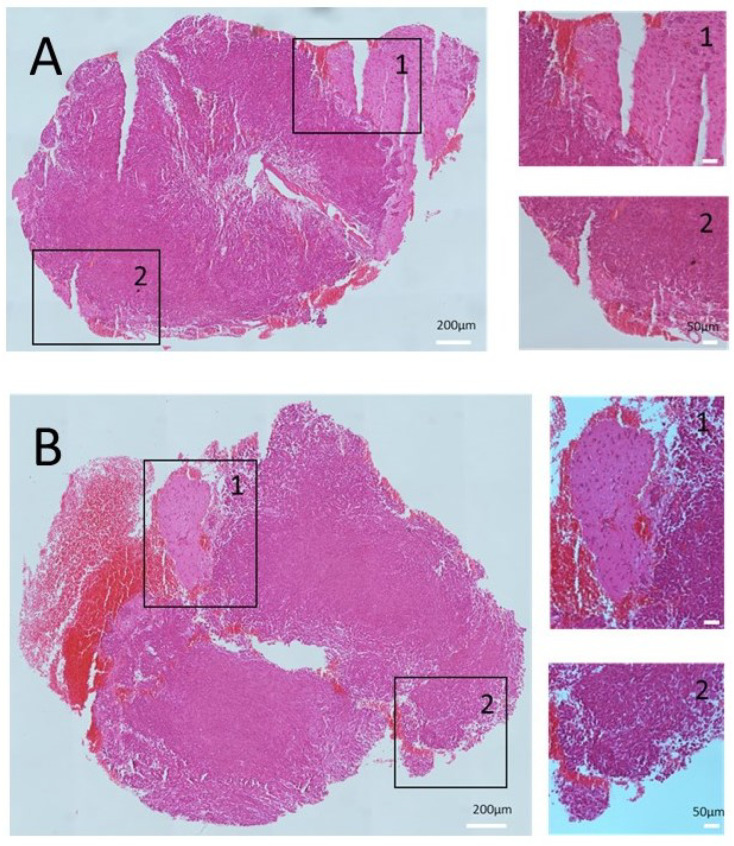
Histological photos of a complete (**A**) and incomplete (**B**) resected tumor, with inserts (1 & 2) showing the margins at higher magnifications. Resected primary tumors were fixed in 4% paraformaldehyde in PBS for 48 h and subsequently processed, resulting in formalin-fixated paraffin-embedded tissue blocks. Then, 7 μm sections were subjected to H & E staining following standard procedures.

Histology confirmed tumor growth after tumor cell injection and recurrence after tumor resection in all rats. Evaluation of the resected tumor margins showed complete resection in all but one tumor (see Appendix A Figure A1). In the first cohort tested (*n* = 6), carprofen via water did not reach the recommended plasma level. The second cohort (*n* = 8) therefore received pre-surgical carprofen in water and one post-surgical injection. Repeated anesthesia via intraperitoneal injection of chloral hydrate did not cause any signs of ileus or peritonitis, as was confirmed upon opening the abdominal cavity for transcardial perfusion.

### 3.2. Clinical Score and Body Weight

**Figure 2:**
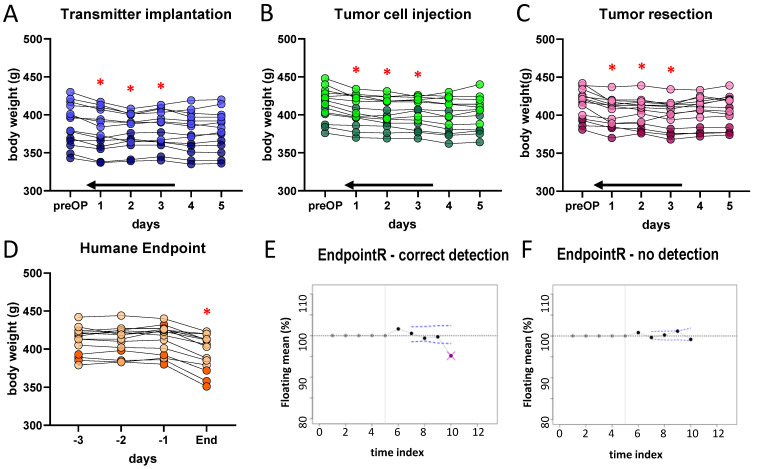
The body weight before and after surgical interventions and in relation to humane endpoint detection. Specifically, the figure displays preoperative body weight and the individual values from five postoperative days following transmitter implantation (**A**, blue), tumor cell injection (**B**, green), and tumor resection (**C**, pink), as well as the body weight on the three days before humane endpoint detection (**D**, orange). The data are presented as individual weight measurements for the first (*n* = 6 dark colors) and second cohorts (*n* = 8 light colors). Perioperative carprofen treatment until postoperative day 3 is indicated by arrow. Statistically significant differences compared to the respective preoperative weight or the day of humane endpoint detection are indicated as asterisks (* *p* < 0.05, post hoc comparison following a significant ANOVA). Panels (**E**,**F**) depict a typical example of the weight trajectory of an individual rat with successful (**E**) and unsuccessful (**F**) warning for humane endpoint determination using the endpointR tool (version 0.0.1.9000). Black dots indicate the normalized body-weight trajectory, blue dashed lines the upper and lower tolerance limits, magenta crosses the days flagged as a warning, and the horizontal and vertical dotted lines the 100% baseline and the start of the evaluation window, respectively.

Except for one rat that showed mildly reduced activity (score 2) for two days post-resection, the clinical score was not affected by any surgery. We examined whether the perioperative BW differed between the first and second cohort, which slightly differed in the pain management regimen. Across the entire observation period (i.e., also pre-surgery), BW was consistently higher in the second cohort compared to the first cohort (two-way RM ANOVA, factor “cohort”: all F > 9.899, all *p* < 0.008). In both cohorts, mean BW declined modestly after each surgical procedure: 2.2% on average (maximum 4.2%) after transmitter implantation, 2.4% on average (maximum 4.1%) after tumor cell injection, and 3.2% in mean (maximum 5.8%) after tumor resection (*p* < 0.05 post hoc testing following significant two-way RM ANOVA for factor “day”: all F > 17.182, all *p* < 0.001; Figure 2A–C). Analysis of the first three postoperative days showed no beneficial effect of the adapted carprofen regimen. After tumor cell injection or tumor resection, statistical analysis did not show any differences in percentage weight loss between the first or second carprofen cohort (two-way RM ANOVA for factor “cohort”: both F < 0.3218, both *p* > 0.583). After transmitter implantation, weight loss in the second cohort was even slightly but significantly greater than that of the first cohort (second day: 0.7% vs. 3.4%, third day: 0.7% vs. 2.4%; *p* < 0.05 post hoc testing following significant two-way RM ANOVA for interaction between factors “day” and “cohort”: both F > 11.79, *p* < 0.001). The discontinuation of carprofen from postoperative day 3 onward also had no adverse effect. After tumor resection, the second cohort even gained weight between the end of carprofen treatment and postoperative day 5 (*p* < 0.05 post hoc testing after significant two-way RM ANOVA for interaction between factors “day” and “cohort”: F_12,60_ = 3.507, *p* = 0.008; Figure 2C).

On the day of the humane endpoint, rats lost an average of approximately 4.0% of their weight (maximum 7.6%) compared to the three previous days (F_13,39_ = 24.303, *p* < 0.001; Figure 2D). On that day, the endpointR app sent a warning sign in 11 of 14 rats, indicating the need for increased monitoring to detect the first signs of humane endpoint (Figure 2E,F). Clinical scoring showed only a slight postural instability or mild ataxia on the day of humane endpoint.

### 3.3. Voluntary Wheel Running

**Figure 3:**
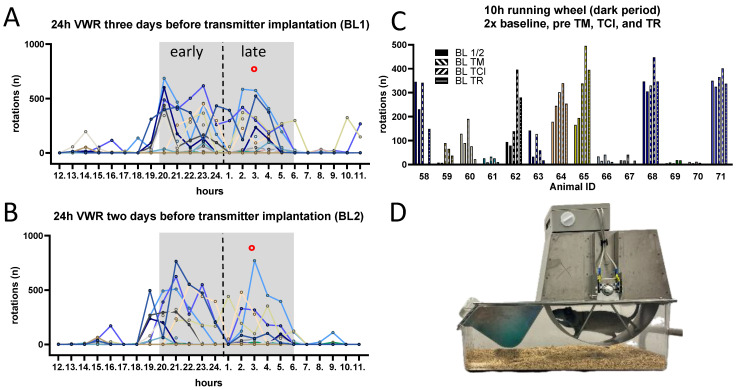
Baseline (BL) voluntary wheel running (VWR) activity three days (BL1; (**A**)) and two days (BL2; (**B**)) prior to transmitter implantation (TM). The data are presented in one-hour bins over a 24 h period for each individual rat. The early and late dark phases of the light/dark cycle are shaded in gray and divided by a dotted line in the early and late dark phases. Statistical analysis revealed that VWR activity was significantly higher in the early compared to the late dark phase (°; *p* < 0.05; post hoc testing following significant ANOVA). The total VWR activity during the dark period at BL1/2, as well as the BL prior to TM, tumor cell injection (TCI) and tumor resection (TR), is shown as a bar graph for each rat in (**C**). The different colors represent the individual animal IDs. The dimensions of the cage setup with the running wheel and a rat are shown as an example ((**D**); brightly lit and without enrichment to improve visibility).

Baseline VWR, analyzed as rotations in 60 min bins across two 24 h periods prior to transmitter implantation, indicated that rats rarely used the wheel during the light phase, with activity mainly occurring after routine inspections in the morning and afternoon (Figure 3A,B). In contrast, rats intensively used the wheel during the dark phase (8:00 p.m.–6:00 a.m.), which was especially pronounced in the early dark phase (8:00 p.m.–1:00 a.m.). We therefore analyzed VWR separately for the early (8:00 p.m.–1:00 a.m.) and late (1:00 a.m.–6:00 a.m.) dark phases. For the baseline recordings three and two days before transmitter implantation, statistical analysis with two-way RM ANOVA with factors “dark phase” and “day” indicated significantly more VWR in the early versus late dark phase (F_1,13_ = 5.862; *p* = 0.031). Although all rats voluntarily used the wheel, qualitative inspection of the VWR revealed substantial inter-individual variability in total VWR across the two baseline recordings. We therefore not only analyzed each rat’s VWR across the two baseline nights but also the nights preceding transmitter implantation, tumor cell injection and tumor resection as additional baseline time points across the experiment. Analysis with one-way RM ANOVA showed that wheel-running behavior differed markedly between individuals but with no obvious alterations over the course of the study (F_4,52_ = 1.640, *p* = 0.178; Figure 3C).

For the perioperative analysis, we excluded rats with missing VWR data on any of the preoperative days or the five postoperative days due to technical issues. This led to the exclusion of one rat after transmitter implantation, three rats after tumor cell injection, two rats after tumor resection, and four rats prior to the humane endpoint, leaving ≥10 rats per condition for analysis (see insets in Figure 3 for details). We confirmed that VWR did not differ between the first and second cohort on the preoperative day or across the five postoperative days (two-way RM ANOVA for factor “cohort”: all F-values < 0.466, all *p* > 0.51; interaction between factor “days” and “cohort”: all F-values < 1.299, all *p* > 0.28).

**Figure 4:**
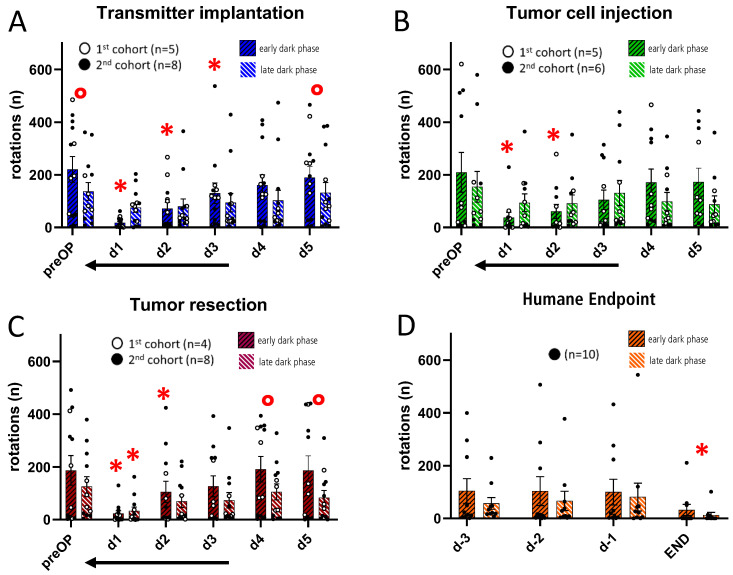
Voluntary wheel running (VWR) activity before surgery (preOP) and across five postoperative days after transmitter implantation (**A**), tumor cell injection (**B**), and tumor resection (**C**), as well as during the three days preceding humane endpoint detection (**D**). Data are shown as individual values and stratified by early versus late dark phase. The number of rats in the first and second cohorts is provided as an inset and is denoted by open and filled circles, respectively. Perioperative carprofen treatment through postoperative day 3 is indicated by the arrow. Significant differences relative to preoperative VWR (**A**–**C**) or relative to the humane endpoint day (**D**) are marked by asterisks (*) for effects within the early or late dark phase, and by circles (°) for differences between early and late dark phases (*p* < 0.05; post hoc comparison following a significant ANOVA).

More detailed analysis of the first three postoperative days also confirmed no differences between the first and second carprofen cohort after surgery for transmitter implantation, tumor cell injection or tumor resection (two-way RM ANOVA for factor “cohort”: all F < 2.167, all *p* > 0.167). After transmitter implantation, VWR was reduced in the early dark phase for three days (post hoc *p* < 0.05 following a significant interaction between factors “day” and “dark phase” F_5,60_ = 8.261; *p* = 0.001). After tumor cell injection, VWR was reduced in the early dark phase for two days (post hoc *p* < 0.05 following a significant interaction between factors “day” and “dark phase” F_5,50_ = 2.600; *p* = 0.036). After tumor resection, VWR was reduced throughout the entire dark phase on postoperative day 1, but was confined to the early dark phase on postoperative day 2 (post hoc *p* < 0.05 following a significant interaction between factors “day” and “dark phase” F_5,55_ = 3.694; *p* = 0.006). Notably, on days 4 and 5 (i.e., after carprofen was resumed), VWR did not differ from preoperative values after either surgery (see Figure 4A–C for details). At the humane endpoint, VWR was significantly reduced with no differences between early and late dark phases (*p* < 0.05 after Friedman test: $\chi^{2}$ = 18.840, df = 3, *p* < 0.001 because of failed normality testing; Figure 4D). At the individual level, all rats showed reduced VWR compared to the previous day.

### 3.4. Activity and Heart Rate Measured with a Subcutaneously Implanted Transmitter

Heart rate and activity recordings began after transmitter implantation; thus, data from one week post-surgery served as baseline. However, in a few rats, the transmitter stopped recording, either because the metal running wheels partially attenuated the transmitter signal or due to other technical issues. This became apparent when we compared dark-phase recordings aggregated in 5 min bins with the VWR data: in a few rats that used the wheel not only for running but also for resting or sleeping, more than 50% of the telemetry data were missing on a given perioperative day. Importantly, because heart rate and activity were recorded independently of the transmitter device, missing values typically occurred in different animals and on different perioperative days.

**Figure 5:**
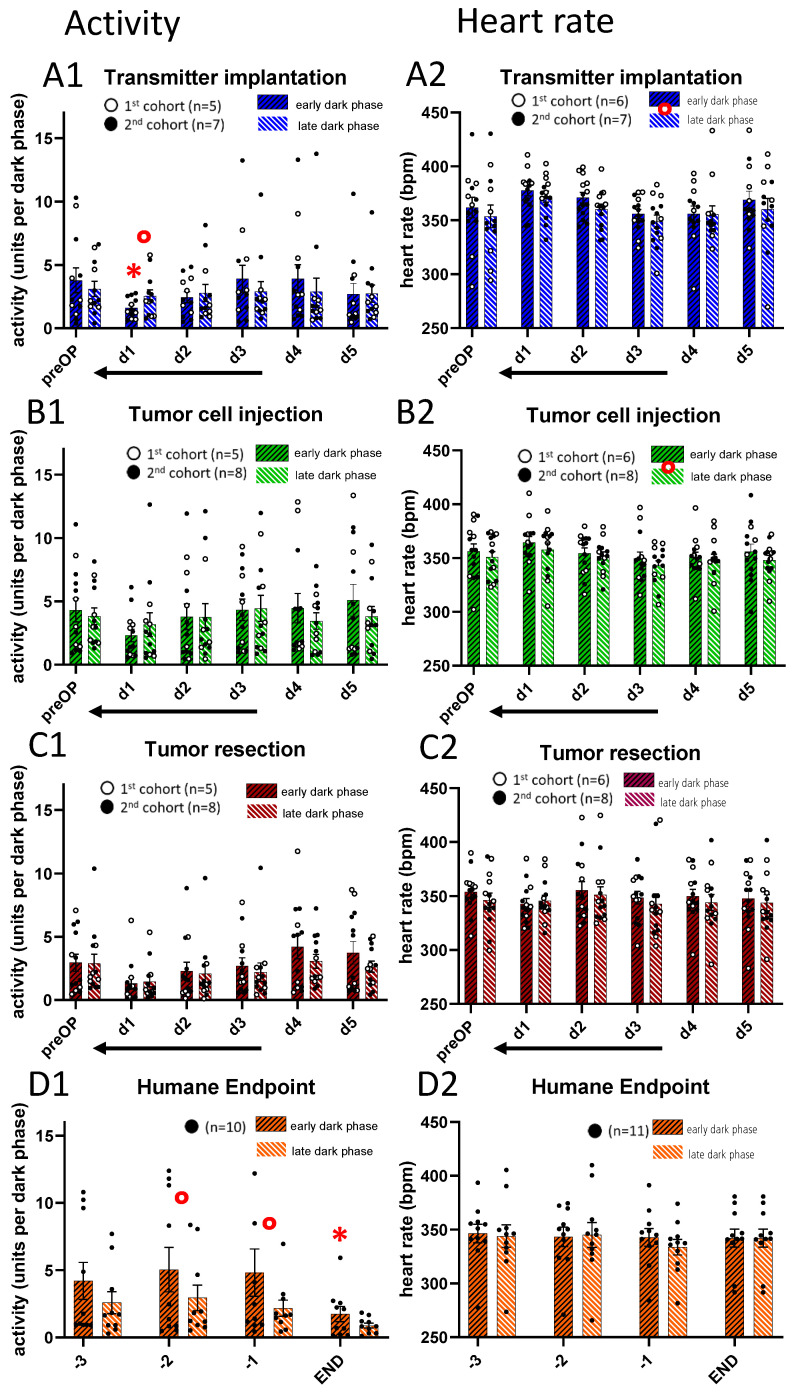
Activity (**A1**–**D1**) and heart rates (**A2**–**D2**) measured by telemetric devices before surgery (preOP) and during five postoperative days following transmitter implantation (**A**), tumor cell injection (**B**), and tumor resection (**C**), as well as three days prior to humane endpoint detection (**D**). The data are displayed as individual values, separated into the early and the late dark phase. Number of rats in the first and second cohorts is shown as insets and represented by open and filled circles, respectively. Perioperative carprofen treatment until postoperative day 3 is indicated by arrow. Significant differences compared to preoperative measures or to the day of humane endpoint detection are indicated as follows: asterisks (*) for significant differences to the preoperative early or late dark phase, and circles (°) for comparisons between the early and late dark phases (*p* < 0.05; post hoc comparison following a significant ANOVA).

Rats with missing activity data on any perioperative day were excluded from the analysis (two rats after transmitter implantation, one rat each after tumor cell injection and tumor resection, and four rats at the humane endpoint), leaving at least ten rats per surgery for analysis. We confirmed that activity measures did not differ between the first and second cohort on the preoperative day or across the five postoperative days (two-way RM ANOVA for factor “cohort”: all F-values < 0.178, all *p* > 0.681; interaction between factor “days” and “cohort”: all F-values < 1.141, all *p* > 0.350). Similar to VWR, activity decreased after surgery, but mostly without significance and with smaller differences between early and late dark phases. Activity was only significantly reduced the day after transmitter implantation in the early dark phase (post hoc *p* < 0.05 following significant two-way RM ANOVA for interaction between factors “day” and “dark phase”: F_5,65_ = 3.648; *p* = 0.006; Figure 5A1). Tumor cell injection and tumor resection had no effect on activity. Activity on days 4 and 5 (i.e., when carprofen was resumed) did not differ from preoperative values after either surgery (see Figure 5A–C for more details). At the humane endpoint, two-way RM ANOVA showed significance for dark phase (F_1,27_ = 5.158, *p* = 0.049) and “days” factors (F_3,27_ = 4.426, *p* = 0.012). Post hoc pairwise comparisons indicated that, on the day of humane endpoint detection, activity was reduced primarily during the early dark phase relative to the preceding days (*p* < 0.05; Figure 5D1). However, at the individual level, only 8 of the 10 rats showed reduced activity values. After transmitter implantation, recording of heart rate failed in one rat on two postoperative days and at the humane endpoint in three rats. These rats were therefore excluded from analysis. Heart rate of the remaining rats did not differ between the first and the second cohort on the preoperative and 5 postoperative days for any surgery approach (two-way RM ANOVA for factor “cohort”: all F-values < 1.27, all *p* > 0.29). Heart rate was enhanced in the early compared to the late dark phases in the perioperative phase of transmitter and tumor cell injection surgery (two-way RM ANOVA for factor “dark phase”: F > 6.176; *p* < 0.027), but not affected by any surgery approach or humane endpoint (Figure 5A2–D2).

### 3.5. Comparison of Surgery and Humane Endpoint

**Figure 6:**
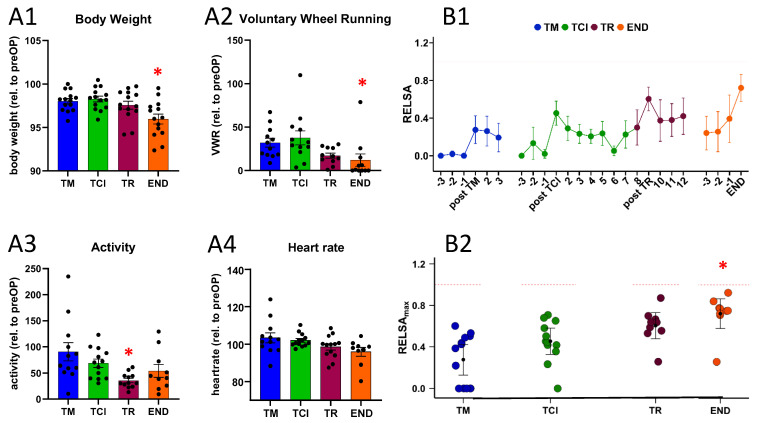
The individual values measured during the dark phase for body weight (**A1**), voluntary wheel running (**A2**), activity (**A3**), and heart rate (**A4**) relative to preoperative values obtained prior to transmitter implantation (TM), tumor cell injection (TCI), and tumor resection (TR), as well as to the day before humane endpoint detection (END). Significant differences compared to TM and TCI are indicated by asterisks (*; *p* < 0.05; post hoc comparison following a significant ANOVA). The RELSA_*flow*_, as the average RELSA over time, shows the relative severity of the experimental groups, standardized to three days before and multiple days after the intervention, up to the humane endpoint. The analysis shows increasing severity with each additional treatment, culminating at the endpoint (**B1**). The RELSA_*max*_ indicates the maximum relative severity achieved by each animal during the observation period. A linear trend of severity from TM to END shows increasing maximum severity with each additional treatment up to the endpoint (**B2**).

Compared to the previous day, rats lost more weight by the day of the humane endpoint compared to that after transmitter implantation and tumor cell injection (*p* < 0.05 post hoc testing after one-way ANOVA: F_3,52_ = 5.750; *p* = 0.002), whereas tumor resection showed a trend (*p* = 0.069; Figure 6A1). Likewise, VWR was more reduced on the day of humane endpoint compared to that after transmitter implantation and tumor cell injection (*p* < 0.05 post hoc testing after Kruskal–Wallis ANOVA because of significant Shapiro–Wilk: H_3_ = 15.886, *p* = 0.001; Figure 6A2). Notably, on the day of the humane endpoint, all rats showed clinical signs of postural instability or mild ataxia accompanied by reduced VWR and weight loss. Activity was more reduced after tumor resection than after transmitter implantation or tumor cell injection (*p* < 0.05 post hoc testing after Kruskal–Wallis ANOVA: H_3_ = 12.449; *p* = 0.006; Figure 6A3). Heart rate did not differ between surgery and the humane endpoint (Figure 6A4). The RELSA_*flow*_ visualizes only mild severity after transmitter implantation and tumor cell injection, while severity increased after tumor resection before peaking at the endpoint (Figure 6B1). Likewise, the RELSA_*max*_ did not differ between transmitter implantation and tumor cell injection, was higher after tumor resection, and highest at the humane endpoint (*p* < 0.05 compared to transmitter implantation and tumor cell injection; Figure 6B2).

## 4. Discussion

In a rat model of intracranial glioma, home cage wheel running enabled continuous, observer-independent grading of postoperative severity and disease progression toward humane endpoint. All rats voluntarily engaged with the running wheel. The majority of activity occurred during the early dark phase—which is similar to the behavior of mice. During the light phase, wheel use was rare and mostly observed following husbandry procedures, suggesting that wheel running may also act as a coping mechanism [28]. Despite pronounced individual differences, each animal exhibited stable running patterns, consistent with previous reports [29] with no obvious changes throughout the study. Individual differences in VWR did not depend on social hierarchy, since all animals were single-housed. Notably, although some rats used the running wheel very little, these reduced measurements still allowed us to extract a decrease in postoperative activity, particularly a reduced use of the running wheel on the day of the humane endpoint. Consequently, the exclusion of rats exhibiting low wheel-running activity, as implemented in other studies [22], should be made with caution.

During the postsurgical recovery, VWR allowed grading of the postoperative burden of intervention complexity. After implantation of a subcutaneous transmitter and after injection of tumor cells into the frontal cortex via a small burr hole, VWR was initially reduced, but normalized to baseline measures by the late post-surgical dark phase. In contrast, VWR was significantly reduced for the entire dark phase after a large craniotomy and tumor removal within the frontal brain parenchyma. Previous studies have reported mild and transient weight loss of less than 5% after tumor cell injection and even tumor resection [24]. Notably, a complex multimodal assessment approach using principal component analysis of clinical scores, activity measures, species-specific behaviors, and home cage video analysis revealed no substantial changes after intracranial tumor cell injection. In contrast, tumor resection via large craniotomy resulted in clear cluster separation, which was even more pronounced on the day of humane endpoint detection [30]. This was mainly driven by squinting and back arching, i.e., behaviors typically associated with pain [31,32,33,34]. The home cage VWR method may therefore be a sensitive and routinely applicable approach to support the determination of burden after surgery. As VWR is also sensitive to analgesic side effects, it may also be a useful measure to improve postsurgical pain management [22]. We further showed that VWR is valuable as a supportive parameter for weight measurements and clinical scoring in the context of humane endpoint detection. Although a weight loss of more than 20% is often used to define a “severe” state, fast-growing intracranial tumor models typically exhibit a prolonged asymptomatic period despite significant tumor size before a sudden increase in intracranial pressure. In our intracranial glioma model, this results in an abrupt 3–5% weight loss accompanied by a clinical decline, which requires euthanasia, as the condition quickly deteriorates. However, a major challenge in defining humane endpoints is ensuring reliability not just at the group level but for each individual animal. While behavioral tests like burrowing and motor coordination were impaired at a group level, not every rat was affected. In contrast, all animals exhibited weight loss alongside declining clinical scores on the endpoint day [24].

To improve objectivity, we previously developed an advanced BW algorithm that functions as a predictive warning system for determining humane endpoints. EndpointR calculates daily, individualized thresholds based on recent weight trends, which accurately identify humane endpoints with up to 97% accuracy [24]. However, this algorithm does not function as a strict criterion but rather as an alert system to identify days that require increased attention and close inspection of the animal. Final euthanasia decisions are still based on clinical scoring, which is brief and susceptible to the expertise and subjective interpretation of the observer. Notably, in the present study in addition to slight weight loss, all rats showed reduced VWR the night before reaching humane endpoints, which may serve as an additional, observer-independent parameter to support or challenge clinical scoring following alerts from the endpointR app. However, in the present study, the weight trajectory algorithm issued warnings in only ∼80% of cases, possibly due to the large craniotomy mitigating sudden intracranial pressure increases from tumor regrowth. Still, reduced wheel running and mild weight loss triggered closer monitoring, ultimately leading to euthanasia. Notably, one animal reached its endpoint unexpectedly early after tumor resection, indicated by weight loss, a mildly impaired clinical score, and reduced VWR. Histological analysis of the resected tumor indicated incomplete removal and accelerated tumor regrowth. This highlights the added value of VWR and weight tracking for early detection, especially when clinical signs are still subtle and disease progression deviates from typical patterns.

Together, VWR is a sensitive and easily accessible proxy for burden after neuroscience intervention, influenced by stress, pain, and mobility. When interpreted with model-specific knowledge, the combination of BW and home cage-derived VWR enables timely, context-dependent decisions, such as euthanasia at the humane endpoint, supporting more effective and ethical animal care. Notably, this is also supported by the outcome of RELSA_*flow*_ and RELSA_*max*_ using BW and VWR as parameters.

In contrast, neither heart rate nor activity measurements obtained via subcutaneously implanted transmitters were as effective as BW or VWR in distinguishing between different surgical interventions and identifying humane endpoints. While activity levels produced comparable but less intense results than VWR or BW, heart rate remained unaffected by any procedure. However, a notable limitation was the interference between transmitters and running wheels, as the metal structure of the wheels disturbed the recording of heart rate and/or activity data whenever a rat was inside the running wheel. Missing data mainly affected the days immediately preceding, or coinciding with, the humane endpoint. However, as noted above, measurements at this stage of the experiment should be interpreted on an individual-animal basis. While VWR was reduced in all rats at the humane endpoint, overall activity was reduced in only ∼80% of animals.

Several limitations need to be addressed with regard to our results. The use of a metal running wheel heavily interfered with the acquisition of transmitter data in some animals. The resulting attrition rate reduced the final sample size available for analysis. As a consequence, the results should be interpreted with caution due to potentially reduced statistical power and possible selection bias. Therefore, a final assessment of the suitability of transmitter data for burden analysis should rely on experimental setups without running wheels or other metallic enrichment that may disturb the transmitter signal. Furthermore, our analyses to date provide no indication that telemetry measures of heart rate or activity are superior to wheel-running data with regard to burden assessment. In addition to being technically vulnerable in enriched cage environments, a telemetry device requires an additional surgical intervention. Its use for routine evaluation within an experiment should therefore be carefully considered. Instead, VWR may offer a non-invasive and more easily implemented measure, which can be regarded as enrichment that can additionally generate valid data for burden assessment once the technical issues are further minimized. Nevertheless, the rats’ activity may have been focused on wheel running, resulting in less observable activity within the cage itself. Another limitation is that the activity measures recorded by the transmitter did not allow differentiation between locomotor activity and other activity measures potentially related to discomfort, such as scratching. In this regard, we observed only qualitatively, and in selected cases, that scratching did not increase activity measures. Furthermore, because transmitter-based monitoring did not allow individual rats to be tracked in group housing, rats were single-housed. Although they were not completely socially isolated, which has been shown to affect VWR [35], they had visual, olfactory, and auditory contact with other rats. Nevertheless, single housing may have affected their welfare and behavioral baseline, as rats are highly social animals. Accordingly, the “control” baseline in single-housed rats may already have been shifted and may not be directly comparable to that of group-housed animals [36]. Thus, although VWR may be a useful enrichment and monitoring tool, its welfare value depends on the housing context in which it is implemented. In future studies, the VWR of individual rats should be tracked using RFID chips, which would then allow group housing. Furthermore, only male rats were used for this study because of the restraints of our animal facility, although differences between males and females with regard to VWR or activity have been reported before [37,38]. Sex differences in VWR are well established and could affect baseline running and postoperative trajectories [39]. Finally, our initial findings showed that adapting our carprofen regimen in the second cohort had no beneficial effect on postoperative body weight or VWR activity. However, these data were collected in the context of an ongoing experiment designed to assess the potential use of VWR as a measure of severity following intracranial surgery. This resulted in the absence of dedicated control groups and relatively small subgroups, which limits the statistical power. Additionally, the study did not employ pain-specific behavioral readouts. Therefore, firm conclusions regarding the analgesic efficacy of the carprofen regime cannot be drawn at this stage.

## 5. Conclusions

We demonstrated that home cage VWR is a sensitive, supportive method for assessing the duration and extent of postoperative burden in male BDIX rats following subcutaneous and intracranial surgeries of varying complexity under the housing conditions studied here. While VWR provided valuable input for determining humane endpoints, final decisions still depended on body weight, clinical score, and closer inspection of the animals, based on prior knowledge of the model. VWR may therefore serve as an effective, easily implemented refinement tool for home-cage-based welfare and severity assessment. As an additional routine parameter, it could improve monitoring in future studies, but broader generalization requires further validation in neuroscience models, including females, different strains, and group-housed conditions.

## Data Availability

The datasets generated during and/or analyzed during the current study are available from the corresponding author upon reasonable request due to ethical restrictions related to the underlying animal experiment data.

## Abbreviations

The following abbreviations are used in this manuscript:

ANOVA	Analysis of variance
BL	Baseline
BW	Body weight
DFG	Deutsche Forschungsgemeinschaft
DSI	Data Sciences International
END	Humane endpoint detection
LAVES	Lower Saxony State Office for Consumer Protection and Food Safety
PBS	Phosphate-buffered saline
RELSA	Relative Severity Assessment
RELSA_*flow*_	Time-dependent RELSA score
RELSA_*max*_	Maximum RELSA score
RFID	Radio-frequency identification
RM	Repeated measures
SEM	Standard error of the mean
TCI	Tumor cell injection
TM	Transmitter implantation
TR	Tumor resection
VWR	Voluntary wheel running

## Appendix A

**Table A1:** Clinical scoring.

Clinical Score	Appearance and Behavior	Instructions
Score 1	active, strong and fast movements, normal exploratory behavior	
Score 2	active with some interruptions of activity, exploratory behavior	
Score 3	prolonged lack of activity, limited exploratory behavior upon external stimuli, mild ataxia with intact muscle tonus	Observation twice daily *
Score 4	ataxia, no exploratory behavior, severely limited reaction to external stimuli, decreased muscle tone, hunched posture, ruffled fur, delayed righting reflex	Humane endpoint
Score 5	no righting reflex, premortal stage	Humane endpoint

* sudden weight loss and mild ataxia (score 3) are humane endpoint criteria for the end of the experimental course, as previous studies have shown that this combination reliably predicts rapid deterioration and death, most likely within the following hours.
